# **Changes in portal blood flow and liver functions in cirrhotics during Ramadan fasting in the summer; a pilot study**

**Published:** 2016

**Authors:** Salem Y Mohamed, Mohamed H Emara, Hala IM Hussien, Hany M Elsadek

**Affiliations:** 1 *Internal Medicine Department, Faculty of Medicine, Zagazig University, Egypt*; 2 *Tropical Medicine Department, Faculty of Medicine, Zagazig University, Egypt*

**Keywords:** Cirrhosis, Ramadan Fasting, Portal vein, Congestion Index

## Abstract

**Aim:**

Assessment of short term changes in portal blood flow and long term changes in liver functions in cirrhotic patients who chose to fast during the month of Ramadan in summer.

**Background:**

During Ramadan, healthy Muslims obligated to fast from predawn to sunset.

**Patients and methods:**

Forty cirrhotic patients intended to fast during the month of Ramadan in the year 2014, were examined by Congestion index (CI) as a non-invasive indicator of short term changes in the portal blood flow, while liver function tests were determined as an indicator of long term changes in liver functions.

**Results:**

A total of 38 patients completed the whole month fasting and two patients discontinued fasting due to variceal bleeding. The complicated patients were 7. CI showed a statistically significant increase from fasting to postprandial status (P<0.001), with statistically significant increases from fasting to postprandial status in Child class A (P<0.001), and B (P<0.001). We did not find a statistical significance between patients with complications and those without complications (P=0.6). There was a statistically significant rise in the serum bilirubin after Ramadan. Deterioration noticed as advanced Child classes, development of lower limb edema, increasing ascites, increasing jaundice and overt encephalopathy.

**Conclusion:**

Cirrhotic patients showed significant short-term changes in the portal blood flow. However, these changes are not linked to complications or deterioration of liver functions and accommodated especially in patients with Child class A and B. Child class C patients should not fast.

## Introduction

 Ramadan is a sacred month for Muslims all around the world. During this lunar-based month, healthy adult Muslims are obligated to abstain from eating, drinking, smoking, or using oral medications from predawn to sunset. Followers will typically eat just after sunset and again before dawn (1). The Islamic lunar calendar is 11 days shorter than the Gregorian solar calendar, and therefore, the month of Ramadan can occur in any season of the year. The hours spent on fasting can vary from 12 to 18 h, depending on the seasonal and regional features (2)

The impact of fasting during the holy month of Ramadan on Muslim patients with various diseases is under extensive discussion in the medical literature, including patients with chronic liver disease. However, the existing data about chronic liver disease in the literature are scarce and give inconclusive results. Compliance with medications may be an issue, especially for those on daily daytime dosages. Also, postprandial hyperemia cannot be accommodated by the liver circulation because of endothelial dysfunction, resulting in a marked postprandial increase in portal pressure. Therefore, action plans must be individualized to enable patients with these conditions to fast without adverse events (3, 4). 

Patients with liver cirrhosis are at risk of many complications during fasting, particularly for protein-energy malnutrition, which is a risk factor for poor prognosis. The risk of upper gastrointestinal bleeding (UGIB), deterioration of liver functions, and development of hepatic encephalopathy (HE) were also reported (5, 6) Consequently, patients should be monitored closely by their physicians while fasting. If any signs/symptoms of liver deterioration occurred, the fasting should be permanently discontinued (6).

This pilot study aimed to assess short-term changes in portal blood flow and long-term changes in liver functions in cirrhotic patients during Ramadan fasting through the hottest month of the year. 

## Patients and Methods

During the year 2014, the lunar month of Ramadan occurred during the summer time in the Northern hemisphere and it began from the late June through July. During this time the atmosphere was hot and dry with average daytime temperature around 35C◦ and the day time was more than 14 hrs. This study was conducted in Tropical and Internal Medicine Departments, Zagazig University Hospitals, Sharkia Governorate, Egypt. Patients were recruited from both the outpatients, as well as inpatient wards. Congestion index (CI) was calculated in the fasting and postprandial status as a non-invasive indicator of short term changes in the portal blood flow. Liver enzymes, as well as liver and kidney function tests were determined before and after Ramadan as an indicator of long term changes in the liver functions in cirrhotics after Ramadan fasting during the summer.

**Definitions**

- Short term changes in portal blood flow: changes in the portal blood flow during fasting and after Iftar including one day of Ramadan.
- Long term changes in liver functions: change in the liver function within one week before Ramadan and one week after Ramadan.
- Iftar: The first diet the followers receive, at the time of sunset.
- Sohor: The last diet followers receive in the late night at the dawn time.
- Complicated patients: patients who experienced deterioration or new manifestations from the baseline at the beginning of Ramadan.

**All patients were subjected to:**

- Thorough history taking.
- Complete physical examination with special emphasis on the stigmata of chronic liver diseases.
- Battery of laboratory investigations, including: Complete blood count, kidney function tests, liver function tests (serum albumin, bilirubin, ALT, AST, Prothrombin time and concentration), viral markers (HBs Ag, HCV antibodies) and autoimmune markers (ANA, SMA, AMA), as well as AFP Abdominal ultrasonography for assessment of liver size, echogenicity, focal lesions, and presence of ascites and/or splenomegaly, as well as determination of portal vein patency.
- Doppler ultrasonography on the portal vein was performed using real time ultrasound with 3.5 Mhz transducer and a pulsed Doppler device (Medison, 8000 EX, Korea). The patients were examined at 12 pm to represent the fasting status and 2 hours after iftar to represent the postprandial status. Breath was held during the examination; and portal vein (PV) diameter was measured. The examination was done with the smallest angle can be taken between the axis of ultrasonic beam and the longitudinal axis of the vessel. For each measurement, at least three reproducible spectral patterns were recorded for calculating the maximum portal blood velocity (Vmax) over a period of 3-4 second to ensure accuracy. The mean portal blood velocity (Vmean) was calculated by the equation [Vmean=0.57×Vmax]. Cross-sectional area (cm^2^) was also recorded at the site of the main portal vein where the portal blood velocity was measured. Congestion index (CI) was calculated by the equation [CI = (area/Vmean) ×100], which was about 0.071 ± 0.014 in normal subjects (7).
- Medical therapy: A reduction in drug compliance was an inherent negative aspect of fasting. Consequently, patients were kept in the best supportive care, including silymarin 140 mg/BID, low dose diuretics, lactulose, Vitamin K supplementation, and the specific therapy when needed. In addition, medications time was adjusted after Iftar and Sohor except for diuretics, which were taken after Iftar (early night) to minimize daytime exhaustion.
- Dietary advice: Frequent meals and snacks can reduce the muscle breakdown between meals, and improve nitrogen balance. A bedtime snack is critical to reduce the breakdown of lean muscle mass during the overnight fast. Small, frequent meals also address the early satiety that many patients experience. Oral liquid supplements have been shown to help increase calorie and protein consumption, and may allow faster gastric emptying than a solid meal (8). Consequently, patients were kept on strict dietary advice to insure best nutritional status and avoid catabolic state. They were instructed to postpone Sohor till the time of dawn and take Iftar just with sunset and keep taking adequate oral fluids in between, with a midnight snack. Meanwhile, they were recommended to stay at home during day time and avoid direct exposure to the sunlight. They were prescribed a sachet form of branched chain amino acid once at night.
- Patients were instructed for emergency admission in the hospital if they developed any complications, including bleeding, encephalopathy, fever, increasing ascites or any major health problem. Patients who developed complications were admitted to the hospital and permanently discontinued fasting.

**Exclusion criteria**

We excluded patients who were not eligible to fast either due to the advanced stage of liver disease, complicated cases or other co-morbidity that interferes with fasting (Four patients with Child class C insisted on fasting by their own good, so they were included in the study). Also, patients on any drug that can affect portal vascular bed excluded from the study.

**Ethical approval**

The study protocol was approved by the Institutional Review Board of the Faculty of Medicine, Zagazig University, Egypt. All patients provided written informed consent to participate in the study and perform all relevant interventions.

**Statistical analysis**

Data were collected, entered, and analyzed using SPSS version 15 (SPSS Inc., Chicago, Illinois, USA) for data processing and statistics. Data were expressed as number and percentage with analysis by Wilcoxon signed rank test for paired qualitative variables and as mean±SD with analysis by paired-t test for quantitative variables. A P-value of less than 0.05 indicated statistically significant results. 

## Results

**Basal characteristics of patients**

The baseline characters of the studied patients are shown in table 1. Out of the 40 patients studied; 38 (95%) completed the whole month fasting and only two (5%) patients discontinued fasting due to the development of variceal bleeding while fasting. These two patients discontinued fasting after 12 and 15 days of fasting. 

**Table 1:** Demographic, clinical and laboratory data of the patients

Parameters				Fasting patients (n=40)
Sex		Male		36(90)*
		Female		4(10)
Age (Years)				59.9±8.9 (44-80)†
Residence (urban/rural)				12/28 (30/70)
Etiology	HCV			28(70)
	HBV			4(10)
	Autoimmune			2(5)
	Mixed			2(5)
	Cryptogenic			4(10)
Hemoglobin (g/dl)				11.1±1.5 (6.9-13.4)
White Blood Cells (x10^3^/mm^3^)				5.2±2.1 (2.2-12)
Platelets (x10^3^/mm^3^)				119.2±64.7 (20-258)
Alanine transferase (IU/L,10- 40 )				50.1±22.5 (17-90)
Albumin (g/L)				3.6±0.45 (2.9-4.8)
Prothrombin concentration (%)				73.4±22.9 (20-100)
Bilirubin (mg/dL)				1.1±1 (0.25-4.8)
Creatinine (mg/dL)				1.0±0.3 (0.8-2.2)
Ultrasonography			Classic	30(75)
			Hepatic focal	4(10)
			Ascites	4(10)
			Focal lesions and ascites	2(5)
Ascites (clinical)			No	34 (85)
			Mild	2(5)
			Moderate	4(10)
Encephalopathy			Grade II	2(5)
Child class			A	26(65)
			B	10(25)
			C	4(10)

* Number (percent);

† Mean± standard deviation (range)

The whole complicated patients (during and after fasting) were 7 out of the 40 patients (17.5%). Most cases were cirrhotics of Child A due to HCV infection. Minority of cases had moderate ascites (10%), grade II HE (5%), and hepatic focal lesions consistent with hepatocellular carcinoma (10%) at the beginning of fasting.

**Portal vein hemodynamics**

Congestive index showed statistically significant increase from fasting to postprandial status as a whole (0.25±0.18 Vs. 0.3±0.17, t test 8.3, P<0.001, data not shown),with a statistical significant increase from fasting to postprandial status in Child class A and B (0.2±0.1 Vs. 0.25±0.1, t test 7.1, P <0.001 and 0.25±0.1 Vs. 0.29±0.1, t test 12.3, P <0.001 respectively), while, Child class C patients showed statistically non-significant increase in the congestive index, (0.45±0.2 Vs. 0.47±0.2, t test 0.8, P value 0.9 data not shown). When we expressed the postprandial increase in the congestive index as a percentage from the fasting status, we did not find a statistical significance between patients with complications (7 patients) and those without complications (33 patients) [median and range, 20% (0.0-57.1%) Vs. 11.1% (4.3-85.7%), Mann-Whitney test 100.5, P value 0.6 (data not shown).

**Liver functions and fasting**

Most of laboratory parameters showed no statistically significant difference after fasting Ramadan apart from a statistically significant rise in the serum bilirubin after Ramadan. The deterioration noticed in some liver functions after the end of Ramadan represented by a statistically significant shift towards more advanced Child classes after fasting in the studied patients. This manifested in the form of developing lower limb edema, increasing ascites, increasing jaundice and development of overt HE. Although, all these items did not show significant changes by the end of the month (Table 2).

Regarding clinical and laboratory parameters, hemoglobin and white blood cells showed a statistically significant reduction in Class A (P=0.01, 0.03, and 0.04 respectively), while platelets showed statistically significant reduction in Class A and B (P=0.04 and 0.008, respectively), after Ramadan. Liver function tests did not show statistically significant changes among different Child classes apart from statistically significant deterioration in serum albumin and bilirubin in Child class C(P= 0.04 and 0.01 respectively), while, serum creatinine showed statistical significant increase in Child class B only (Table 3). 

## Discussion

Based on Islamic principles, patients are exempted from fasting during Ramadan. Those, whom fasting may aggravate their conditions, are exempted. However, each year many Muslim patients express their willingness to observe the fast during Ramadan to respect the traditionals.

There are concerns regarding the impact of starvation, dehydration, and change in the dietary style and discontinuation of medications during Ramadan fasting for patients with liver diseases. This concern is particularly important when Ramadan coincides with hot, dry summer and long days. This is confirmed to be deleterious for renal patients, although cirrhotic patients who are at risk of renal dysfunction (9). In our study, we tried to control the previous factors through the given instructions to the patients as we previously mentioned. 

Cirrhotic patients’ management becomes even more difficult when Ramadan falls during the northern-hemisphere summer, because the period of fasting is longer, and the 2 meals are eaten closer together. Also, meal ingestion causes a physiological increase of splanchnic blood flow, which is associated with an acute increase in hepatic venous pressure gradient (HVPG) in patients with cirrhosis (10, 11). 

In our study, out of 40 patients, 38 patients (95%) completed the whole month fasting and only 2 patients discontinued fasting due to the development of variceal bleeding. The whole complicated patients were 7 out of the 40 patients (17.5%). The percentage is smaller than previously reported (5, 6) of 32.6% and 20.4%, respectively. This may be due to the small number of patients, most of them with an early Child score or due to heath education and nutritional support for the patients included in the current study.

**Table 2:** Clinical and laboratory data of patients before and after fasting

Parameters		Before Fasting (n=40)	After Fasting (n=40)	Paired t-test	P
Hemoglobin (g/dl)		11.1±1.5	11.2±1.6	0.4	0.7
WBCs (x10^3^/mm^3^)		5.2±2.1	5.3±2.3	0.22	0.82
PLTs (x10^3^/mm^3^)		119.2±64.7	111.5±54.2	1.5	0.12
Alanine transferase (IU/L) (10- 40 )		50.1±22.5	54.3±19.5	1.5	0.12
Albumin (g/L)		3.6±0.45	3.5±0.5	1	0.3
Prothrombin (%)		73.4±22.9	75.3±17.5	0.8	0.4
Bilirubin (mg/dL)		1.1±1	1.5±1.1	3.2	0.002
Creatinine (mg/dL)		1.0±0.3	1.1±0.6	1.6	0.1
Ascites	No	34 (85%)	30 (75%)	1.2*	0.26
	Mild	2(5%)	4(10%)		
	Moderate	4(10%)	6(15%)		
GI bleeding	once	0	2(5%)	1.4*	0.15
Encephalopathy		2(5%)	4(10%)	1.4*	0.15
Child class	A	26(65%)	24(60%)	2*	0.04
	B	10(15%)	10(15%)		
	C	4(10%)	6(15%)		

* Wilcoxon signed rank test.

**Table 3:** Clinical and laboratory data of patients before and after fasting among different Child classes

	Child A (n=26)				Child B (n=10)			Child C (n=4)		
	Before	After	P	Before		After	P	before	after	P
Hb	11.47±1.2	11.60±1. 4	0.31	10.48±2.2		10.48±2. 2	1	11.15±0.5	10.55±0.6	0.002
WBCs	5.45±2. 2	5.21±2.1	0.03	5.40±1.9		6.02±2. 9	0.13	3.35±1.3	3.65±0.4	0.56
PLTs	144.38±64.2	131.00±55.1	0.04	84.40±26.9		74.60±33.6	0.008	45.0±28.8	77. 0±10.4	0.20
ALT	53.79±21.3	57.69±20.9	0.21	46.60±29		50.60±17.4	0.50	39. 0±1.2	41.5±4.0	0.18
Alb	3.76±0.2	3.69±0.4	0.24	3.38±0.8		3.51±0. 8	0.18	3.25±0.5	2.85±0.2	0.04
P.conc	77.50±25.2	83.26±10.3	0.16	71.70±14.6		68.60±19.8	0.16	51.25±10.1	44.50±2.9	0.15
Bil	0.91±0.3	0.94±0.3	0.07	1.73±0.9		1.80±0. 8	0.18	3.70±1.3	4.00±1.2	0.01
Cr	0.93±0.02	1.12±0.13	0.11	0.92±0.1		1.02±0.1	0.001	1.5±0.7	1.7±0.5	0.18
Ascites	0	2	1.6	2		4	0.06	4	4	1.0
UGIB	0	0	1.0	0		0	1.0	0	2	1.5
HE	0	0	1.0	0		0	1.0	0	2	1.5

Hb=hemoglobin, WBC s= white blood cells, PLTs =platelet count, ALT=alanine transferase, P.conc=Prothrombin concentration, Bil= bilirubin in mg/dl, cr= creatinine in mg/ dl,UGIB=upper GI bleeding, HE=hepatic encephalopathy.

Two large studies (5,6) from Egypt focused on the effect of Ramadan fasting on liver functions and conducted nearly at the same time but at different centers. The first study was a single center study and focused on both cirrhotic and chronic hepatitis patients and found that dyspepsia and UGIB were more pronounced in the fasting group. Specifically, cirrhotic patients showed deterioration of liver functions in the form of increasing Child class C patients after the month of Ramadan. Noticeably, the risk of variceal bleeding was little in fasting cirrhotic patients compared to non-fasting cirrhotics (6). The second study was a multi-center study and involved 300 cirrhotic patients and found that BMI, serum glucose, ALT, AST, GGT, and ALP decreased; while serum bilirubin increased significantly after full Ramadan fast and they concluded that patients with Child A liver cirrhosis and with no history of GI bleeding may tolerate fasting Ramadan. However, older patients with Child C cirrhosis associated with GI bleeding or diabetes mellitus should be advised not to fast (5).

To the best of our knowledge, the current study is the first that focused on changes in portal hemodynamics by Ramadan fasting. In the present study, Congestive index showed statistically significant increase from fasting to postprandial status as a whole, with a statistical significant increase from fasting to postprandial status in Child class A and B with the probability of increased portal flow and consequently the risk of bleeding is present, this increased portal congestion by Doppler study was proved in earlier studies to be a predictor of upper gastrointestinal bleeding (12), although, Child class C showed non-significant statistical increase in the congestive index. However, the portal congestion index can be affected by several factors such as; portal venous pressure, portal vascular resistance in the liver, portal blood flow, and the development of Porto systemic collateral pathways (7). When we expressed the postprandial increase in congestive index as a percentage from the fasting status, we did not find a statistical significance between complicated patients and non-complicated pointing to other contributing factors, as previous studies shown that the conditions of feeding imposed by Ramadan are associated with an increase in gastric acidity and pepsin activity mainly in diurnal phase(13), which may be a risk factor that can cause bleeding or rebleeding from peptic ulcer (14). This means that modifications in gastric acid secretion are likely to increase the risk of upper gastrointestinal bleeding during Ramadan (10, 11). It has been previously reported that the time distribution of food intake during Ramadan has a metabolic effect, especially a daily increasing in gastric acidity and peptic activity(13). Furthermore, Ramadan fasting led to a decrease in the platelet responses of different aggregating agents in vitro in normal healthy subjects. It also led to an increase in bleeding time and coagulation time(15). 

Our study revealed that most of the laboratory parameters showed no statistically significant difference apart from a statistically significant rise in the serum bilirubin. Although, it was reported that glucuronosyltransferase genetic polymorphisms may be associated with liver cirrhosis (16), which may be affected by fasting (17). However, we thought that this significant rise is part of the general deterioration of the hepatic functions as it was in Child class C and was associated with reduced serum albumin in the same class. Also, this class had 2 patients with UGIB, which may aggravate this deterioration and follow by spontaneous bacterial peritonitis. The deterioration noticed in some liver functions after the end of Ramadan in our patients was represented by a statistically significant shift towards more advanced Child classes after fasting in the studied patients. This significant shift manifested in the form of developing lower limb edema, increasing ascites, increasing jaundice and development of HE. Although, all these items did not show significant improvement by the end of the month. 

 The rest of liver function tests did not show statistically significant changes among different Child classes, while serum creatinine showed statistically significant increase only in Child class B. 

Malnutrition in patients with cirrhosis is prevalent and is found in 25% of patients with Child class A and up to 80% in patients with Child class C (18). Most cirrhotic patients have an inadequate caloric intake due to multiple factors, including early satiety related to impaired gastric relaxation and ascites, as well as poor appetite. Furthermore, many of them have nutrient malabsorption due to portal hypertension (18). Moreover, due to poor capacity of the liver to store glucose in the form of glycogen, the cirrhotic patient switches to gluconeogenesis from amino acids after an overnight fast. This is observed in non-cirrhotic patients only 3 days after fasting. We tried to hinder this phenomenon by giving the patients a midnight snack, night BCCA, and delaying the Sohor to the dawn time. 

In their study, Yamanaka-Okumura, et al.(19) showed that a late evening snack prevented the deterioration in subjective measures of global health and mental health at 6 months in patients with cirrhosis and is reinforced by our results, most of our patients showed non-significant reductions in serum albumin levels and little deterioration in the other parameters.

The role of nutrition as one of the most important factors that can influence overall mortality and morbidity in end stage liver disease has been well understood and appreciated. Without any doubt, liver cirrhosis drives the patients to a catabolic state, depriving them of essential nutrients. This would explain the statistical significant increase of serum creatinine in Child class B within the normal range, though mechanisms that need to be well understood by physicians. This was not obvious in Child class C due to a marked reduction in muscle bulk, which gives the patient what known by a spider appearance with the presence of ascites (20). Therefore, in our study, we decided to supply patients with night BCCA, and this was confirmed a long time ago in a study by Fukushima, et al. (21), who reported that night BCCA supplementation is beneficial for the liver than morning supplies and this was confirmed in another study as well (22). Additionally, there are recommendations for patients with cirrhosis to have five meals spread throughout the day to prevent a negative nitrogen balance and possibly help with their cognitive function. Thus, we advised our patients to take Iftar and Sohor; as well as a midnight snack besides adequate fluids.

Patients with liver cirrhosis on diuretic therapy with long daytime of water deprivation may be at risk of dehydration and renal shut down, although was not confirmed in this study. We instructed the patients to consume accepted amounts of fluids, especially plain water during the non-fasting hours. This is reflected by non-significant increases in serum creatinine level as a whole and we did not have an acute kidney injury (23) even in patients with UGIB. Also, we did not report cases of hepatorenal syndrome during the study period.

With focus on each Child class, we found that hemoglobin showed statistical significant reduction in Class C, which can be understood as 2 patients in this class had UGIB. Little consensus exists regarding blood count outcomes during Ramadan. The majority of studies found that hemoglobin levels do not change(24), but decreased levels have also been reported (25, 26). In our study, white blood cells showed statistically significant reduction in Class A, without a constant pattern in the other classes, we could not interpret these results because of many untested reasons; the possibility of bacterial infections or hypersplenism, while platelets showed statistical significant reduction in Class A and B, after Ramadan with non-significant change in Child class C may be due to a small number of the patients or the markedly reduced platelet count. However, bad dietary habits (that may be practiced before fasting) had been incriminated in this issue as it was reported that a relationship between the type of diet and chronic low-grade inflammation, an underlying pathophysiological mechanism linking behavioral factors and oxidative stress to the risk of developing chronic disease (27).

This study has some limitations. First, it is a single center study conducted in Egypt with temperate atmosphere when compared with zones nearer to the equator with more rise of temperature and humidity. Second, the small number of patients recruited. Third, lack of control group. The reason was the pilot purpose of the study, a larger study to confirm the results of the current study with a control arm is needed. Fourth, the lack of studying multiple confounding factors of the results. For example; the effect of portal decompressive drugs and the presence or absence of portal collaterals and esophageal varices on the congestive index were not studied. Finally, Child classification to determine the deterioration noticed in liver functions, with some variables subjective and inter-observer variability. However, it uses only some lab parameters that are done routinely. 

In conclusion, cirrhotic patients showed significant short term changes in the portal blood flow, however these changes were not linked to complications or deteriorations of liver functions and can be accommodated especially in patients with Child class A and B. Therefore, they can fast with good medical and nutritional care. Since fasting has been associated with complications and deterioration of liver functions in the long-term event, patients with Child class C should be prohibited from fasting
